# Abstracting Dance: Detaching Ourselves from the Habitual Perception of the Moving Body

**DOI:** 10.3389/fpsyg.2017.00776

**Published:** 2017-05-16

**Authors:** Vered Aviv

**Affiliations:** The Jerusalem Academy of Music and DanceJerusalem, Israel

**Keywords:** dance perception, human motion analysis, performing arts, movement prediction, abstract art

## Abstract

This work explores to what extent the notion of abstraction in dance is valid and what it entails. Unlike abstraction in the fine arts that aims for a certain independence from representation of the external world through the use of non-figurative elements, dance is realized by a highly familiar object – the human body. In fact, we are all experts in recognizing the human body. For instance, we can mentally reconstruct its motion from minimal information (e.g., via a “dot display”), predict body trajectory during movement and identify emotional expressions of the body. Nonetheless, despite the presence of a human dancer on stage and our extreme familiarity with the human body, the process of abstraction is applicable also to dance. Abstract dance removes itself from familiar daily movements, violates the observer’s predictions about future movements and detaches itself from narratives. In so doing, abstract dance exposes the observer to perceptions of unfamiliar situations, thus paving the way to new interpretations of human motion and hence to perceiving ourselves differently in both the physical and emotional domains.

## Introduction

Abstraction is a fundamental process in arts, such as in music and in the fine arts. In the latter (primarily in painting), this process is largely understood. There is a general consensus that abstract visual art presents non-figurative elements to the observer’s visual system. These elements serve as vehicles for the artists to express their ideas, for instance on the relationships and tensions between these abstract elements. Exposing the viewer to a visual scene without identifiable objects, the visual attention and range of associations that are evoked in the spectator’s mind are likely to be different from those elicited when looking at recognizable objects (Augustin et al., 2008; Aviv, 2014; Chatterjee and Vartanian, 2014; Schepman et al., 2015).

However, unlike the fine arts (and music) the notion of abstraction is not obvious when considering dance. Indeed, dance is mediated by the human body (the dancer), which is a highly familiar object. Humans are experts in analyzing the movements of the human body, predicting its future trajectory and understanding the functional aspects of an action and its emotional content. Hence, is it at all possible to attribute the notion of “abstraction” to dance that is mediated by such a familiar and concrete object – the human body?

The term abstraction was actually referred to dance by various contemporary as well as past choreographers and dance researchers, typically illuminating the intention of the choreographer to create dance without a narrative [e.g., Oskar Schlemmer’s work in Lahusen (1986); William Forsythe’s work in Nugent (2007)]. However, the present article focuses on the viewer’s experience rather than on choreographer’s intention. It asks to what extent could the viewer perceive dance as being “abstract” albeit the dominant presence the dancer?

This article argues that it is indeed possible and useful to consider the process of abstraction in dance. It starts by presenting several definitions of the general process of abstraction in both the fine arts and in science. Next, based on behavioral and neurobiological studies, it examines human motion in terms of perception, prediction, and empathy. Finally, the article shows why some features are candidates for abstraction in dance while other features are not fit for abstraction. Clarifying the process of abstraction in dance provides tools for dance analysis and is therefore useful for researchers, as well as choreographers and dancers in their creative process.

## On Abstraction

*Abstraction is a way of acquiring knowledge about the world* (Zeki, 2000).

It is generally assumed that abstraction is a built-in process typical to people’s way of thinking (see Root-Bernstein, 2001; Zimmer, 2003; Gray and Tall, 2007; Henriksen et al., 2014). The processes of categorization and classification such as grouping a dancer, a child, and a man sitting in a chair under the category of “human” inevitably involve abstraction, because they seek to preserve certain features shared by these objects while ignoring other features of these individuals. From this point of view, all art, including figurative painting, implements some degree of abstraction because it is never an accurate representation of reality *per se* (Gortais, 2003; Zimmer, 2003). Gray and Tall (2007) suggested that the abstraction process is governed by the compression of knowledge into thinkable concepts, which yields a more sophisticated way of apprehending these concepts. In other words, abstraction is the process of building thinkable concepts from a situation which can readily be accessed by more elaborate ways of thinking and understanding. Several authors have argued that abstraction is a multilayered process, based on the ability to focus on key features as well as to find analogies between seemingly dissimilar entities (**Figure 1A** and Henriksen et al., 2014).

**FIGURE 1:**
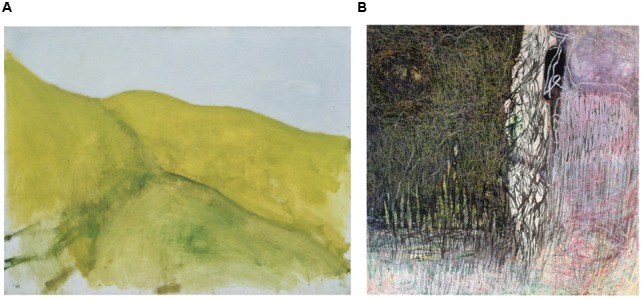
**Levels of abstraction in the visual arts. (A)** An example of figurative or semi-figurative painting illustrating some of the characteristics of abstraction in art, such as selection (of gross forms only, and using a limited palette of colors), elimination (of pictorial details), amplification (of the forms such as silhouettes and the contours between them), and the analogy between dissimilar entities (the female body and a landscape); in Ori Reisman, Woman-landscape, 1960s, Oil on canvas, 130 cm × 97 cm, courtesy of the Reisman collection, Kibbutz Cabri, Israel. **(B)** Fully abstract painting, eliminating any figure or object representation, focusing only on the medium as means of expressions of ideas. Zvi Mairovich, Already More Near Than Far, 1966/68, Panda, 100 cm × 100 cm, Courtesy of S. Mairovich.

There is a general consensus that the process of abstraction, in particular in science and in art, involves selection and elimination. During abstraction, some elements of the target of abstraction are chosen to remain (selection), and can also be amplified, whereas other components are partially or totally eliminated (**Figure 1** and Zeki, 2000; Root-Bernstein, 2001; Zimmer, 2003; Levins, 2006; Gray and Tall, 2007; Henriksen et al., 2014).

Because abstraction always involves choices of what is selected and how much is eliminated, it is clear that there is more than one way to abstract the target of abstraction. This holds in both science and in art (Zimmer, 2003; Levins, 2006). The strength or meaningfulness of the abstraction can be assessed by the extent by which it preserves and emphasizes the key components of reality (in science) or the visual reference in the world (in the visual arts), leading to new insights and understanding of the subject matter (Levins, 2006; Henriksen et al., 2014). The strength of an abstraction is a subjective measure because its assessment depends on the community (e.g., scientists or artists), their interests, context and perspective. In science, researchers have argued that a productive abstraction should eventually lead to a theory and not just to a description of a phenomenon as such (Levins, 2006). Below I use the concepts of selection, amplification and elimination to examine abstraction in dance. A comprehensive discussion of the notion of “abstraction” from philosophical, cultural, psychological, artistic and scientific, perspectives could be found in Langer (1953) and Toscano (2008).

## Abstraction in the Visual Arts

Observing abstract visual art reveals some of its specific characteristics. One of these features is that abstract visual art exposes the viewer’s visual system to an unusual situation, in which the visual scene is made up of unrecognizable objects that escape categorization (**Figures 1B, 2C**) and therefore is likely to evoke different (“object-free”) associations as compared to that invoked by a figurative painting such as in **Figure 2A** (Kawabata and Zeki, 2004; Vartanian and Goel, 2004; Aviv, 2014). Another important feature of abstract visual art is that the basic means of expression (such as the brush traces on the canvas or the thickness of the paint) can become the main message, as opposed to compositions of objects (the “content”) in figurative art (Gortais, 2003; Zimmer, 2003; Belke et al., 2006). An interesting behavioral outcome of abstract visual art is the way that the visual scene is scanned by the observer. Whereas in figurative art the observer’s gaze is focused mostly on salient recognizable elements in the scene (**Figures 2A,B**), in abstract art the viewer’s gaze wanders more uniformly over the whole pictorial scene, which is related to the level of abstraction (Pihko et al., 2011; Taylor et al., 2011, see **Figure 2C**). **Figures 2A,B** demonstrate that when a human figure is present visually, the eyes are automatically fixated on the salient, highly recognizable, features of the person. This is the case in both the fine arts and the dance.

**FIGURE 2:**
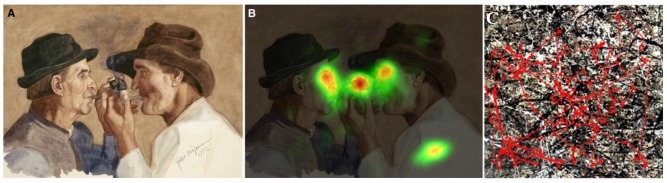
**Differences in eye scanning patterns of a figurative versus abstract painting. (A)** Figurative painting, “Piipunsytyttäjät/Lighting pipes” by Rissanen (1902). **(B)**. Eye tracking distribution of 20 individuals on this painting. Note the focal distribution of the fixation points that concentrates primarily on the faces, the pipe and the artist’s signature (replicated from Hari and Kujala, 2009). **(C)** Fixation (red lines) distribution on an abstract painting by Jackson Pollock. Note the broad trajectories of the eye tracking movements in the case of abstract art (replicated from Taylor et al., 2011).

Interestingly, eye-tracker studies on art perception have demonstrated that, albeit variability between viewers, there are common patterns of fixation on an art work. Such patterns include fixating more on sharp versus none-sharp resolution and longer fixations on human and animals versus other non-human figurative elements such as clouds. These studies reveal that even in two dimensional representational paintings of human figures, the gaze of the viewer will examine more the limbs of a “dynamic figure” (indicating a person in motion) rather than a “static figure” (a non-moving figure) (Quiroga and Pedreira, 2011; Massaro et al., 2012).

## Our Expertise in Analyzing of Human Motion and Dance

Humans are highly sensitive to and are experts in analyzing human motion (Blake and Shiffrar, 2007). It was shown that people are able to extract important information just by looking at a partial implied movement, such as on a point-light display animation, whereby a small number of light spots are positioned on several major joints of a person (Johansson, 1973); see https://www.youtube.com/watch?v=f8TFi6qvPbc\&index=1\&list=PLE2CA19BBD7BB8EF5. Analyzing the pattern of motion of these light points presented against a dark background (without seeing the person), people can realize the shape of the person (tall, pregnant), their sex, determine and the nature of activity (dancing, walking, see, Dittrich, 1993; Cutting and Kozlowski, 1977; Pollick et al., 2005; Sebanz and Shiffrar, 2009). People can also grasp the emotional state of the animated person to a significant extent (anger, sadness, or happiness, see for example Dittrich et al., 1996; Chouchourelou et al., 2006; Ikeda and Watanabe, 2009; Alaerts et al., 2011). From point-light display animation we can also intuit properties of the objects handled by people, such as the object’s weight or elasticity (Bingham, 1993; Clarke et al., 2005; Blake and Shiffrar, 2007). All the above examples demonstrate that the human visual system is highly skilled at comprehending another person’s movements and actions, and in mentally reconstructing the body’s motion and its action from very limited information. Importantly, based on past experience, the motion-detection visual system generates predictions about future motion. This creates a surprise when a dancer performs an unexpected movement and satisfaction, when our expectations are met (Blakemore and Decety, 2001; Hagendoorn, 2004; Kilner et al., 2007; Blasing et al., 2012).

Several studies have shown that understanding other people’s movements is based substantially on one’s own experience in movement planning and execution. We are more accurate and some of our brain’s motor areas respond more strongly when we watch movements of another person that we have executed in the past (Calvo-Merino et al., 2005, 2006; Cross et al., 2006; Blake and Shiffrar, 2007; Karpati et al., 2015). While watching another person moving, we may experience partial recruitment of our own sensory and motor representations, presumably via the proposed mirror neuron system (and action observation network). The latter coordinates the motion we see performed by another person with the corresponding sensorimotor representations elicit when we move (Gallese et al., 1996; Rizzolatti and Craighero, 2004; Rizzolatti and Sinigaglia, 2010). These data illustrate another major quality of visual perception of motion: watching a human in motion recruits not only the visual system but also the motor system of the observer (motor representation, motor planning, motor prediction, Blakemore and Decety, 2001; Jeannerod, 2004; Blake and Shiffrar, 2007; Karpati et al., 2015; Philbeck and Witt, 2015).

Another aspect of human motion perception is that it is tuned to identify the sociological information portrayed by the movements of the other. This can involve understanding another person’s intentions by looking at his action (e.g., watching someone raising his hands and assuming he plans to hug the person in front of him) or comprehending the emotional state of the performer (e.g., perceiving him as being calm) by watching his movements. There is now a better understanding of the brain areas involved in interpreting the information conveyed by the body with reference to the intentions and emotions of the other, including brain areas involved in decoding the meaning of compound themes such as expressions of hope or exhaustion (see Blakemore and Decety, 2001; Tipper et al., 2015). One can distinguish between movement intention (the type of movement that will be executed, such as a turn) and the intention of the action (the goal of the movement such as throwing a disk) (Ondobaka et al., 2012). It has been suggested that people attribute intention to another person’s actions by mentally simulation of the consequence of such action in their own sensorimotor system (see Blakemore and Decety, 2001; Tipper et al., 2015).

Emotions (emotional expression) transmitted via the body (to be distinguished from the more specific facial expressions or hand gestures) are identified rapidly, and we can reliably differentiate several types of emotions (De Gelder, 2006; Dael et al., 2012). Dance researchers have shown that trained dancers, in comparison to non-dancers, are more accurate at discriminating the emotions expressed in dance, and they are also more responsive at the level of their own physiological arousal to the expressed emotions. Nevertheless, both groups, dancers and non-dancers, recognize and identify which emotions are expressed in the dance. Furthermore, the emotional valence seems to be transmitted through the quality of the movement and not through a particular sequence of movements or steps (see Christensen et al., 2016)

All of the above constitute strong evidence for people’s specially developed mechanisms to watch, identify, and understand human motion. This is also pertinent to observing dance. The impact of our advanced capabilities to analyze human (and biological) motion implies that when we watch a dancer, we cannot avoid analyzing all these qualities: the dancer’s identity (sex, age, personal identity), movements, actions, intentions, and emotions. This may appear to suggest that there cannot be abstraction of the human figure during dance, but rather a concrete practically automated and elaborated perception and interpretation of a moving dancer.

## Which Aspects of Dance Could be “Abstracted”?

A key facet of abstract in fine arts, the usage of non-figurative elements as the building blocks of composition is inherently absent in dance, because of the presence of the human body. This fundamental difference may imply that the notion of “abstract dance” is an oxymoron. However, it is posited here that, from the observer’s point of view, certain *components* associated with the process of abstraction can exist in dance.

Dance is a multifunctional socio-cultural event and a multidimensional phenomenon comprising many components, i.e., motion, narrative or semi-narrative scenes, music, costumes, lighting, stage design and others (Jola et al., 2012). This article focuses on the prospect of abstraction of the main and essential component of dance – the motion of a dancer. Other non-movement components of dance are also candidates for abstraction, first and foremost music. Their synergetic effect, when taken together, on the perception of a certain dance as being abstract should be thoroughly investigated. This, however, is beyond the scope of the present article.

Dance can be stripped from the associated narrative and many of the above mentioned non-movement components, from obvious goal directed movements and from functional as well as meaningful actions and gestures (such as clasping another dancer, holding onto a chair, etc.). Once dance is pared down to purposeless, functionless movements one can start discussing abstract dance. The automatic cognitive prediction of upcoming movements can be circumvented by performing non-stylized motions (as opposed to movements typical to the classical ballet repertoire for example) and by avoiding repetitive movement sequences that are easy to predict after a few exposures. Expectations can also be undermined by generating unusual trajectories that the observer has probably not seen in daily life. Thus, a spectator watching abstract dance would be watching human movement that has no obvious goal and thus would not trigger any clear expectation of the subsequent movement, and would not find an explicit emotion or a specific message conveyed by the movement. Watching a movement that carries a little or no message enables the observer to watch and respond to the course of the movement, rather than to the goal of the movement, as is the usual case in the daily life. The spectator would than observes a movement *per se*, for the sake of motion itself. This is the nearest we can get to abstraction of the art of dance.

I would like to claim that many contemporary choreographers create (either consciously or unconsciously) abstract dances or dance pieces that lack narratives, clear emotional gestures, and introduce unfamiliar movements with trajectories that are often hard to predict. This opens up new range of possibilities for viewers to experience the human body, as well as their own body movements. Perhaps paradoxically, the fact that people have become experts in analysis of the human body in motion, provides new opportunities for abstract dance to suspend what we know about, and expect from, our body and learn something new about ourselves via abstracting dance.

As mentioned above, one of the most significant feature of abstraction in the fine arts is that one observes a representation of the object rather than the object itself. This feature is missing altogether in dance because one is looking at real moving dancer, not on the image of the dancer. Other principles of abstraction such as selection, amplification, and elimination are applied in dance by the selection and restriction of the motion repertoire, and through amplification achieved by increasing the size of gesture, its velocity or its intensity to emphasize and focus on specific expressions. The principle of thinkable concepts can also be applied in abstract dance. For example, when the movements repertoire blurs gender differences, thus highlighting a thinkable concept of “human” as such. Many thinkable concepts that are expressed are not necessarily conscious or verbalized by either the dancer or the spectator, but they shed light on knowledge and general ideas about the human body and its range of movement capabilities.

When we compare the main characteristics of abstract art to abstract dance, there is only a partial overlap. In both abstract art and abstract dance, the main message is transmitted through the raw material of the medium; namely the brush strokes and movements, respectively. For these two types of art, the medium itself is the “message” of the work (the motion itself for its own sake in dance, patches and traces of brush work in plastic art). But whereas abstract visual art creates non-figurative, non-categorized elements in the viewer’s eye, dance always presents human figures to the spectator, with no possible abstraction. While looking at abstract fine art, the spectator’s gaze wanders all over the surface of the canvas, whereas watching a human figure in motion initiates a focal tracking system aimed at the (moving) figure, and very little attention is paid to the background (Wang et al., 2003). Abstract fine art exposes the visual system to an unfamiliar, unusual situation of looking at non-objects, which is likely to introduce different emotional responses and associations (as shown by Lengger et al., 2007; Else et al., 2015). Abstract dance, on the other hand, exposes the spectator to a concrete situation of a moving dancer, but outside of a pragmatic context of predictive and meaningful action. In this sense, there is some resemblance between abstract art and abstract dance, as they both introduce unfamiliar input to the visual system and are therefore likely to evoke uncommon, new responses. It is important to emphasize that, although the criteria proposed hereby for “abstract dance” are universal, the interpretation of a given dance (as being non-abstract or abstract) could be culture-dependent. Indeed, a certain dance scene could evoke specific predictions or convey certain emotions in viewers of one culture (e.g., Western) and not in viewers from another culture (e.g., non-Western), or *vice versa*.

Watching abstract dance can induce different associations and feelings in the viewer which are more dependent on state of mind than a cognitive response to a specific message delivered by gestures. One example is the William Forsythe – “Solo” (1995), made for Evidentia, a program designed by Sylvie Guillem^1^ that reflects on the viewer’s own response to that dance. Other examples are the work “Far” by McGregor (2010) and, to a large extent, the work “Connect Transfer II” by Wei (2008). These works all fulfill the definition proposed herby of abstract dance. They are all wonderful examples of abstract dance and of its powerful effect on our mind.

## Author Contributions

The author confirms being the sole contributor of this work and approved it for publication.

## Conflict of Interest Statement

The author declares that the research was conducted in the absence of any commercial or financial relationships that could be construed as a potential conflict of interest.

## Footnotes

1 https://www.youtube.com/watch?v=hDTu7jF_EwY

## References

[B1] Alaerts K. Nackaerts E. Meyns P. Swinnen S. P. Wenderoth N. (2011). Action and emotions recognition from point light displays; an investigation of gender differences. *PLoS ONE* 6:e20989 10.1371/journal.pone.0020989 PMC3111458 21695266

[B2] Augustin M. D. Leder H. Hutzler F. Carbon C. C. (2008). Style follows content: on the microgenesis of art perception. *Acta Psychol.* 128 127–138. 10.1016/j.actpsy.2007.11.006 18164689

[B3] Aviv V. (2014). What does the brain tell us about abstract art? *Front. Hum. Neurosci.* 8:85 10.3389/fnhum.2014.00085 PMC3937809 24616683

[B4] Belke B. Leder H. Augustin D. (2006). Mastering style. Effects of explicit style-related information, art knowledge and affective state on appreciation of abstract paintings. *Psychol. Sci.* 48 115–134.

[B5] Bingham G. P. (1993). Scaling hile judgement of lifted weigh: lifter size and the role of standard. *Ecol. Psychol.* 5 31–64. 10.1207/s15326969eco0501_2

[B6] Blake R. Shiffrar M. (2007). Perception of human motion. *Ann. Rev Psychol.* 58 47–73. 10.1146/annurev.psych.57.102904.190152 16903802

[B7] Blakemore S. J. Decety J. (2001). From the perception of action to the understanding of intention. *Nature Rev. Neurosci.* 2 561–567. 11483999 10.1038/35086023

[B8] Blasing B. Calvo-Marino B. Cross E. Jola C. Honisch J. Stevens C. J. (2012). Neurocognitive control in dance perception and performance. *Acta Psychol.* 139: 300–308. 10.1016/j.actpsy.2011.12.005 22305351

[B9] Calvo-Merino B. Glaser D. E. Grezes J. Passingham R. E. Haggard P. (2005). Actin observation and acquired motor skills: an F-MRI study with experc dancers. *Cereb. Cortex* 15 1243–1249. 10.1093/cercor/bhi007 15616133

[B10] Calvo-Merino B. Grèzes J. Glaser D. E. Passingham R. E. Haggard P. (2006). Seeing or doing? Influence of visual and motor and familiarity in action observation. *Curr. Biol.* 16 1905–1910. 10.1016/j.cub.2006.07.065 17027486

[B11] Chatterjee A. Vartanian O. (2014). Neuroaesthetics. *Trends Cogn. Sci.* 18 370–375. 10.1016/j.tics.2014.03.003 24768244

[B12] Chouchourelou A. Matsuka T. Harber K. Shiffrar M. (2006). The visual analysis of emotional actions. *Soc. Neurosci.* 1 63–74. 10.1080/17470910600630599 18633776

[B13] Christensen J. F. Gomila A. Gaigg S. B. Sivarajah N. Calvo-Merino B. (2016). Dance expertise modulates behavioral and psychophysiological responses to affective body movement. *J. Exp. Psychol. Hum. Percept. Perform.* 8 1139–1147. 10.1037/xhp0000176. 26882181

[B14] Clarke T. J. Bradshaw M. F. Field D. T. Hampson S. E. Rose D. (2005). The perception of emotion from body movement in point-light displays of interpersonal dialogue. *Perception* 34 1171–1180. 10.1068/p5203 16309112

[B15] Cross E. S. Hamilton A. F. Grafton S. T. (2006). Building a motor simulation de novo: observation of dance by dancers. *Neuroimage* 100 39–50. 10.1016/j.neuroimage.2006.01.033 PMC1821082 16530429

[B16] Cutting J. E. Kozlowski L. T. (1977). Recognizing friends by their walk: gait perception without familiarity cues. *Bull. Psychon. Soc.* 9 353–356. 10.3758/BF03337021

[B17] Dael N. Mortillaro M. Scherer K. R. (2012). Emotion expression in body action and posture. *Emotion* 12 1085–10101. 10.1037/a0025737 22059517

[B18] De Gelder B. (2006). Towards the neurobiology of emotional body language. *Nature Rev. Neurosci.* 7 242–249. 10.1038/nrn1872 16495945

[B19] Dittrich W. H. (1993). Action categories and the perception of biological motion. *Perception* 22 15–22. 10.1068/p220015 8474831

[B20] Dittrich W. H. Troscianko T. Lea S. E. Morgan D. (1996). Perception of emotion from dynamic point-light displays represented in dance. *Perception* 25 727–738. 10.1068/p250727 8888304

[B21] Else J. E. Ellis J. Orme E. (2015). Art expertise modulates the emotional response to modern art, especially abstract: an ERP investigation. *Front. Hum. Neurosci.* 9:525 10.3389/fnhum.2015.00525. PMC4876367 27242497

[B22] Gallese V. Fadiga L. Rizzolatti G. (1996). Action recognition in the pre motor cortex. *Brain* 119 593–609. 10.1093/brain/119.2.593 8800951

[B23] Gortais B. (2003). Abstraction and art. *Philos. Trans. R. Soc. Lond. B Biol. Sci.* 358 1241–1249. 10.1098/rstb.2003.1309 12903659 PMC1693217

[B24] Gray E. Tall D. (2007). Abstraction as a natural process of mental compression. *Math. Educ. Res. J.* 19 23–40. 10.1007/BF03217454

[B25] Hagendoorn I. (2004). Some speculative hypotheses about the nature and perception of dance and choreography. *J. Conscious. Stud.* 11 79–110.

[B26] Hari R. Kujala M. V. (2009). Brain basis of human social interaction: from concepts to brain imaging. *Physiol. Rev.* 89 453–479. 10.1152/physrev.00041.2007 19342612

[B27] Henriksen D. Fanhoe C. Mishra P. (2014). Abstracting as a trans-disciplinary habit of mind. *TechTrends* 58 3–7. 10.1007/s11528-014-0794-x

[B28] Ikeda H. Watanabe K. (2009). Anger and happiness are linked differently to the explicit detection of biological motion. *Perception* 38 1002–1011. 10.1068/p6250 19764302

[B29] Jeannerod M. (2004). Actions from within. *Int. J. Sport Exerc. Psychol.* 2 376–402. 10.1080/1612197X.2004.9671752

[B30] Johansson G. (1973). Visual perception of biological motion and a model for its analysis. *Percept. Psychophys.* 14 195–204. 10.3758/BF03212378

[B31] Jola C. Ehrenberg S. Reynolds D. (2012). The experience of watching dance: phenomenological–neuroscience duets. *Phenomenol. Cogn. Sci.* 11 17–37. 10.1007/s11097-010-9191-x

[B32] Karpati F. J. Giacosa C. Foster N. E. V. Penhune V. B. (2015). Dance and the brain: a review. *Ann. N. Y. Acad. Sci.* 1337 140–146. 10.1111/nyas.12632 25773628

[B33] Kawabata H. Zeki S. (2004). Neural correlates of beauty. *J. Neurophys*., 91 1699–1705. 10.1152/jn.00696.2003 15010496

[B34] Kilner J. M. Friston K. J. Frith C. D. (2007). Predictive coding: an account of the mirror neuron system. *Cogn. Process.* 8 159–166. 10.1007/s10339-007-0170-2 17429704 PMC2649419

[B35] Lahusen S. (1986). Oskar schlemmer: mechanical ballets? *Dance Res*. 4 65–77. 10.2307/1290727

[B36] Langer S. (1953). *Feeling and Form.* New York, NY: Scribner.

[B37] Lengger P. G. Fischmeister F. P. S. Leder H. Bauer H. (2007). Functional neuroanatomy of the perception of modern art: a DC–EEG study on the influence of stylistic information on aesthetic experience. *Brain Res.* 1158 93–102. 10.1016/j.brainres.2007.05.001 17559816

[B38] Levins R. (2006). Strategies of abstraction. *Biol. Philos.* 21 741–755. 10.1007/s10539-006-9052-8

[B39] Massaro D. Savazzi F. Di Dio C. Freedberg D. Gallese V. Gilli G. (2012). When art moves the eyes: a behavioral and eye-tracking study. *PLoS ONE* 7:e37285 10.1371/journal.pone.0037285 PMC3356266 22624007

[B40] Nugent A. (2007). William forsythe, eidos: telos, and intertextual criticism. *Dance Res. J.* 39 25–48. 10.1017/S014976770000005X

[B41] Ondobaka S. de Lange F. P. Newman-Norlund R. D. Wiemers M. Bekkering H. (2012). Interplay between action and movement intentions during social interaction. *Physiol. Sci.* 23 30–35. 10.1177/0956797611424163 22157675

[B42] Philbeck J. W. Witt J. K. (2015). Action-specific influences on perception and post-perceptual processes: present controversies and future directions. *Psychol. Bull.* 141 1120–1144. 10.1037/a0039738 26501227 PMC4621785

[B43] Pihko E. Virtanen A. Saarinen V. M. Pannasch S. Hirvenkari L. Tossavainen T. (2011). Experiencing art: the influence of expertise and painting abstraction level. *Front. Hum. Neurosci.* 5:94 10.3389/fnhum.2011.00094 PMC3170917 21941475

[B44] Pollick F. E. Kay J. W. Heim K. Stringer R. (2005). Gender recognition from point-light walkers. *J. Exp. Psychol. Hum. Percept. Perform.* 31 1247–1265. 10.1037/0096-1523.31.6.1247 16366787

[B45] Quiroga R. Q. Pedreira C. (2011). How do we see art: an eye-tracker study. *Front. Hum. Neurosci.* 5:98 10.3389/fnhum.2011.00098 PMC3170918 21941476

[B46] Rizzolatti G. Craighero L. (2004). The mirror-neurons system. *Ann. Rev. Neurosci.* 27 169–192. 10.1146/annurev.neuro.27.070203.144230 15217330

[B47] Rizzolatti G. Sinigaglia C. (2010). The functional role of parieto-frontal mirror circuit: interpretations and misinterpretations. *Nature Rev. Neurosci.* 11 264–274. 10.1038/nrn2805 20216547

[B48] Root-Bernstein M. (2001). Abstracting bulls: a dancing words/writing dance workshop. *J. Dance Educ.* 1 134–141. 10.1080/15290824.2001.10387194

[B49] Schepman A. Rodway P. Pullen S. J. Kirkham J. (2015). Shared liking and association valence for representational art but not abstract art. *J. Vis.* 15:11 10.1167/15.5.11 26067529

[B50] Sebanz N. Shiffrar M. (2009). Detecting deception in a bluffing body: the role of expertise. *Psychol. Bull. Rev.* 16 170–175. 10.3758/PBR.16.1.170 19145029

[B51] Taylor R. P. Spehar B. Van Donkelaar P. Hagerhall C. M. (2011). Perceptual and physiological responses to Jackson Pollock’s fractals. *Front. Hum. Neurosci.* 5:60 10.3389/fnhum.2011.00060 PMC3124832 21734876

[B52] Tipper C. M. Signorini G. Grafton S. T. (2015). Body language in the brain: constructing meaning from expressive movement. *Front. Hum. Neurosci.* 9:450 10.3389/fnhum.2015.00450 PMC4543892 26347635

[B53] Toscano A. (2008). The culture of abstraction. *Theory Cult. Soc.* 25 57–75. 10.1177/0263276408091983

[B54] Vartanian O. Goel V. (2004). Neuroanatomical correlates of aesthetic preference for paintings. *Neuroreport* 15 893–897. 10.1097/00001756-200404090-00032 15073538

[B55] Wang L. Hu W. Tan T. (2003). Recent developments in human motion analysis. *Pattern Recognit.* 36 585–601. 10.1016/S0031-3203(02)00100-0

[B56] Zeki S. (2000). Abstraction and idealism. *Nature* 404 547–547. 10.1038/35007158 10766218

[B57] Zimmer R. (2003). Abstraction in art with implications for perception. *Philos. Trans. R. Soc. Lond. B Biol. Sci.* 358 1285–1291. 10.1098/rstb.2003.1307 12903671 PMC1693220

